# A comprehensive analysis of selected medicines collected from private drug outlets of Dhaka city, Bangladesh in a simple random survey

**DOI:** 10.1038/s41598-021-04309-1

**Published:** 2022-01-07

**Authors:** Mohammad Sofiqur Rahman, Naoko Yoshida, Hirohito Tsuboi, James Regun Karmoker, Nadia Kabir, Simon Schaefermann, Yoshio Akimoto, Mohiuddin Ahmed Bhuiyan, Md. Selim Reza, Kazuko Kimura

**Affiliations:** 1 Medi-Quality Security Institute (MQS), Graduate School of Medical Sciences, Kanazawa University, Kanazawa, 920-1192 Japan; 2 AI Hospital/Macro Signal Dynamics Research and Development Center, Institute of Medical, Pharmaceutical and Health Sciences, Kanazawa University, Kanazawa, Japan; 3 Department of Clinical Pharmacy and Healthcare Sciences, Kanazawa University, Kanazawa, 920-1192 Japan; 4 Present Address: University of Oklahoma Health Sciences Center, Oklahoma City, OK 73104 USA; 5 Department of Pharmacy, University of Asia Pacific, 74/A Green Road, Farmgate, Dhaka, 1205 Bangladesh; 6 Pharmaceutical Institute, Eberhard Karls University Tuebingen, Tuebingen, Germany; 7 Department of Pharmaceutical Technology, University of Dhaka, Dhaka, 1000 Bangladesh

**Keywords:** Health care, Medical research, Risk factors, Chemistry

## Abstract

Comprehensive data are needed to prevent substandard and falsified (SF) medicines as they pose a major risk to human health. To assess the quality of selected medicines, samples were collected from random private drug outlets of Dhaka North and South City Corporation, Bangladesh. Sample analysis included visual observation of the packaging, authenticity of the samples, legitimacy and registration verification of the manufacturer, physicochemical analysis, and price. Chemical analysis of the samples was performed using a portable Raman spectroscopy and high-performance liquid chromatography according to the pharmacopoeia. Several discrepancies were noted in the visual observation of samples. Among the 189 collected samples of esomeprazole (ESM), cefixime (CFIX), and amoxicillin-clavulanic acid (CVA-AMPC), 21.2% were confirmed to be authentic, 91.3% manufacturers were confirmed legitimate, and 2.1% of all samples were unregistered. Chemical analysis of the samples revealed that 9.5% (95% CI 5.7–14.6) of samples were SFs. Falsified samples and quality variation in the same generic branded samples were both detected by Raman spectroscopic analysis. Overall, sample prices were satisfactory relative to the international reference price. This study documents the availability of poor-quality medicines, demonstrating the need for immediate attention by the national medicine regulatory authority.

## Introduction

Assuring quality in pharmaceutical products is a major public health challenge, requiring an action plan which is capable of mitigating numerous unfavorable factors^1–4^. According to the World Health Organization (WHO), an estimated two billion people around the world do not have access to necessary medicines, vaccines, medical devices (including in vitro diagnostics), and other healthcare products, creating a vacuum that is too often filled by substandard and falsified (SF) medicines^5,6^. The WHO defines substandard medicines, also termed as out of specification, as authorized medical products that fail to meet either their quality standards or specifications, or both. In contrast, falsified medicines are those that deliberately/fraudulently misrepresent their identity, composition, or source^7^. In addition, unregistered/unlicensed medical products are those that have not undergone due evaluation and/or approval by the medicine regulatory authorities (MRAs) for the market in which they are marketed/distributed or used, but are subject to national or regional regulations and legislation^7^.

This issue has been identified as an urgent health challenge for the next decade, given that more than one in ten medicines in low- and middle-income countries (LMICs) are estimated to be SF^8,9^. In recent years, chemical analysis results for select pharmaceuticals also indicated that more inspection and monitoring of medicines is required throughout the Southeast Asia region^10–14^. With the eighth-largest population in the world, Bangladesh has made many developments in the pharmaceutical sector and exports medicines to many countries across the world^15^. Following their first national drug policy in 1982, they were focused on promoting the national drug industry, and they became the first low-income (now lower- and middle-income countries) country to develop an indigenous pharmaceutical industry^16^. Recently, the National Control Laboratory (NCL) for medicinal products in Bangladesh, under the Directorate General of Drug Administration (DGDA), has been recognized as compliant with WHO recommended standards of Good Practices for Pharmaceutical Quality Control Laboratories (GPPQCL)^17^. This is a major step forward towards ensuring that Bangladesh has access to quality essential medicines at the national, regional, and global levels^18^. Approximately 300 pharmaceutical companies in Bangladesh manufacture a variety of medicines, and their products account for 97% of locally-available medicines^19^.

Despite recent reports outlining the issues associated with SF medicines, no systematic survey has been performed in Bangladesh regarding the rampant availability of SF medicines in the country. The current situation is uncertain, and there have been sporadic reports of falsified medicines and their victims, like the paracetamol tragedy reported in 1995^13,19–21^. Although, the actual number was far greater, at least 51 child patients were documented to have died after ingesting a brand of paracetamol elixir. Test results showed that 19 of 69 analyzed paracetamol elixirs from 28 different brands contained diethylene glycol as the sole diluent. During that study period between 1 January 1990 and 1 December 1992, an additional 185 patients with unexplained renal failure died at Dhaka Shishu Hospital, 85% of which had consumed an unknown elixir for fever^21^. In addition, the private retail drug shops market in Bangladesh is largely unregulated and unaccountable, resulting in poor distribution and storage conditions for medicines^22–24^. In this context, there is an urgent need for a preliminary study evaluating the quality of medicines, to identify the prevalence of SFs in the country. Furthermore, there are issues regarding multiple pharmaceutical standards from the same manufacturers, with no justification whatsoever to compromise on the quality of medicines^25^. There have been many reports on the off-site analysis and characterization of SF medicines, including analysis using high performance liquid chromatographic (HPLC) and liquid chromatography-mass spectroscopic methods^3,20^. Despite serious efforts from various stakeholders, methods for the on-site evaluation of SF medicines are scarce and largely elusive; fast on-site detection and analysis methods could empower the surveillance system, providing a first line of defense against SF medicines^26^.

The purpose of the current study was to provide an estimation of the prevalence of SF medicines in private drug outlets in Dhaka city, Bangladesh. Our focus is on prevention, detection, and response, with the aim of achieving increased access to safe, effective, high-quality medical products^27^.

## Methods

### Reporting system and ethical approval

This project was a collaborative effort between Kanazawa University, Japan and University of Asia Pacific, Bangladesh represented by Professor K.K. All the methods in this study were carried out in accordance with relevant guidelines and regulations. The study was conducted and reported according to the Strengthening the Reporting of Observational Studies in Epidemiology (STROBE) guidelines, Medicine Quality Assessment Reporting Guidelines (MEDQUARG), and WHO Guidelines on the conduct of surveys of the quality of medicines^28–30^. The importance of good ethical practice for such studies has been discussed recently by Tabernero et al., to maintain the privacy and confidentiality of the surveyors and the surveyed; however, institutional review board approval was not required for this study at Kanazawa University as it did not involve any live vertebrates, experimental animal and/or human subject research^31^. Regulatory approval was taken in writing from the Directorate General of Drug Administration (DGDA) under the Ministry of Health & Family Welfare, Government of the People's Republic of Bangladesh on the condition that raw data sharing will be limited, and confidentiality with respect to the name of the manufacturers or their commercial brand will be maintained. The final report of this study was submitted to the DGDA in September, 2019.

### Study settings and design

The entire healthcare system of Bangladesh serves an estimated population of 161.03 million, with a density of 1,077 people per square km and a population of the metropolitan area of the capital city Dhaka of approximately 17 million^32–34^. Target medicines were purchased from private retail drug shops (retail shops, wholesale shops selling retail medicines, and a shop in a private hospital located in Dhaka City Corporation of Dhaka District) during April, 2018, without prescriptions, using the mystery buyer approach (Supplemental Fig. S1). A list of shops was obtained from the DGDA and the outlets were classified according to area (Thanas included in the Dhaka North and South City Corporation) and included in the main database. Among the 4400 outlets in Dhaka district listed in the DGDA database, 831 outlets were excluded due to predetermined criteria (for example, Savar, Ashulia). In total, 1,885 outlets from Dhaka North City Corporation (Adabar, Badda, Banani, Gulshan, Kafrul, Khilkhet, Mirpur, Mohammadpur, Pallabi, Shaymoli, Tejgaon, and Uttara) and 1684 outlets from Dhaka South City Corporation (Dhanmondi, Jatrabari, Khilgaon, Kotowali, Motijheel, Paltan, Ramna, Rampura, Shabujbag, and Shahbag) were included in the final list of outlets. The final list of outlets was randomized using a randomization table and the samples were purchased according to the randomized list by four mystery buyer teams over 9 d, including a one-day pre-sampling to demonstrate the sample collection approaches^35^. The required sample size (188) was calculated with a 5% margin of error and 95% confidence levels assuming the sample proportion to be 15%. Target medicines were esomeprazole (ESM), cefixime (CFIX), and amoxicillin-clavulanic acid (CVA-AMPC), and they were chosen based on the frequency of use, availability, and characteristics of the medicines that are included in the essential medicine list of WHO^36^ (ESM as omeprazole).

### Sampling terms

There were four sampling teams, each containing three fourth year Bachelor of Pharmacy students and one supervisor. The samplers and supervisors were trained and instructed on how to purchase the medicines. Samples were purchased by selecting at least one sample of either ESM, CFIX, or CVA-AMPC from the visited outlets over 9 d. Variations in the generic brands and strength depended on the availability of the samples in the outlet during the purchase. In principle, the primary approach was to collect 100 dosage units per sample (not less than 40 units per sample in case of unavailability). Medicines collected from the same outlet, labeled with the same international nonproprietary (INN) name, brand name, strength, size, batch/lot number, and manufacturing and expiry dates were considered as one sample. The outlet type, date of purchase, price paid, brand name, formulation, batch number, date of manufacture, and expiry date were recorded using a standard sampling form for every sample purchased. Every sample was placed in an individual ziplock bag together with the recoded data and securely stored in an air-conditioned room (20–25 °C) until analysis.

### Sample analysis

Collected samples were shipped within one month to the analytical laboratory at Kanazawa University for analysis. Sample analysis consisted of visual examination of the samples (packages, strips, and tablets/capsules), an investigation of the authenticity of the product by the manufacturer, verification of the legitimacy of manufacturers, registration verification of the product in Bangladesh by DGDA, Raman scattering, and pharmacopoeial analysis. Details of the packaging condition and label information were recorded carefully, according to the tabulated checklist. During observation, the packaging and labeling, physical appearance of the tablet/capsule, batch number, manufacturing date, and date of expiration, were examined according to the WHO guidelines on the conduct of surveys of the quality of medicines and the International Pharmaceutical Federation (FIP) checklist for visual inspection of medicines^37,38^.

For confirming the authenticity, a detailed questionnaire was sent to each manufacturer to confirm the authenticity of the product. Each questionnaire provided detailed information about the product, including manufacturer, batch number, date of manufacture and expiry date, dosage, and strength of the product, as recommended by the WHO^29^. Verification of the legitimacy of the manufacturers and the registration status of each product was evaluated by visual inspection of the packaging, and then by sending a questionnaire along with the package and photographs of the sample to the DGDA to confirm the legitimacy of the manufacturers and the registration of the product^39^.

### Chemical analysis

Laboratory analysis of all the samples was carried out before the expiration date. Pharmaceutical analysis of the samples was performed according to the British and United States pharmacopoeia, with slight modifications as specified in the sample package of the respective dosage form for each of the medicines^40–45^. Some minor adjustments were made to the analytical procedure of ESM and CFX indicated in the pharmacopeia monograph. They are described in the Supplemental File S1. The pharmacopoeial quality assessment included potency (drug content), content uniformity test, and dissolution test.

The reference standards for ESM (as omeprazole), CFIX, amoxicillin, and clavulanic acid, and the internal standards (IS) lansoprazole, metronidazole, and cefadroxil, were all purchased from the United States Pharmacopeial Convention. HPLC-grade acetonitrile and methanol, tetrabutylammonium hydroxide solution ([(CH_3_CH_2_CH_2_CH_2_)_4_N]OH), sodium di-hydrogen phosphate (NaH_2_PO_4_·2H_2_O), di-sodium hydrogen phosphate (Na_2_HPO_4_), tri-sodium phosphate (Na_3_PO_4_·12H_2_O), potassium di-hydrogen phosphate (KH_2_PO_4_), and other chemicals of reagent grade were purchased from Wako (Wako Pure Chemical Industries, Ltd., Osaka, Japan).

For the calibration curves, individual stock solutions were prepared by dissolving reference standards in the corresponding solvent (Supplemental File S1) at a concentration of 0.2 mg/mL. Afterwards, stock solutions were diluted to 5.0, 10.0, 20.0, 30.0, and 40.0 μg/mL aliquots to obtain five calibration samples. The concentration of all IS solutions was 20.0 μg/mL, and these were mixed with the each of the diluted reference standard solutions and sample solutions. For the assay and content uniformity sample solutions were prepared using the same solvent and diluted to the same concentration as the reference standard solutions.

The dissolution test of the samples was conducted with an NTR-VS 6P dissolution apparatus (Toyama Sangyo Co. Ltd., Osaka, Japan), and the assay was carried out by HPLC. The circular dichroism (CD) spectra of the enantiomer of omeprazole (ESM) was detected and measured using a Jasco CD-PDA detector (Chiral detector-CD 2095, and Photo diode array-MD 2018 Plus, Jasco, Tokyo, Japan) equipped with an AS-950 Jasco auto-sampler. The HPLC system for analyzing CFIX and CVA-AMPC samples consisted of a Prominence HPLC equipped with auto-sampler (SIL-10AD) and Ultraviolet-Photo Diode Array Detector (UV-PDA, SPD-20A/20AV Series; Shimadzu, Kyoto, Japan).

Mechanical calibration and performance verification tests were performed before sample testing for performance qualification to ensure the absence of technical and mechanical errors. Test methods and system suitability for each medicine were validated according to USP 41^46^. The five-point calibration curves were prepared with three replicates of injections for each vial of the calibrators, and three replicates were used during sample analysis. Calculations for quantitation were based on the peak area ratios of the analyte relative to its corresponding IS using weighted (1/x) regression. The linearity of the curves was assessed by linear calibration using the correlation coefficient (r). The r was determined using the mean of three replicates at each level of the calibrators. Details of the analytical condition, dissolution test, chromatographic condition, and the reference for compliance criteria are summarized and presented in Supplemental Table S1.

### Sample compliance criteria

In the potency test (quantity), ESM samples were evaluated as meeting acceptance criteria if the amount of API lay within the range of 90.0–110.0% of the label claim. For CFIX samples, the tolerance range was 90.0–110.0% of the label claim. For CVA-AMPC samples, the range was 90.0–120.0% (both amoxicillin and clavulanic acid). For the content uniformity test, the acceptance value was calculated according to USP 41 using Eq. (1)^46^:

1 $$Acceptance \, Value \, \left(AV\right)=\left|M- {\overline{x}}\right|+KS$$

where, ${\overline{x}}$ is the mean of an individual content expressed as a percentage of label claim; *M* is the reference value, *K* is acceptability constant, and *S* is the sample standard deviation.

In the dissolution test, Q values for evaluation were as follows: ESM in acid, not more than (NMT) 10% of the label claim; ESM in buffer, not less than (NLT) Q = 75% of the label claim; CFIX, Q = 75% of the label claim; amoxicillin, = 85% of the label claim; and clavulanic acid, Q = 80% of the label claim.

### Raman scattering analysis of the samples

Raman scattering analysis was performed to analyze the molecular structure by light scattering using a portable Raman scattering analyzer (Inspector 500, SciAps Inc., Laramie, WY, USA). The instrument was equipped with higher wavelength Raman excitation, consisting of a 300 mW power source with a 1030 nm wavelength Class III B laser and a cooled Type III–IV semiconductor detector array (spectral range 100–2500 cm^−1^). The exposure time was set at the default (maximum 8.0 s). Each of the tested samples was analyzed for five consecutive spectral data on the front, back, and side, thus generating 45 spectral data. The average of these 45 spectral data was then calculated and analyzed. Tablet samples were taken out of the blister and kept directly in front of the laser source. For capsule samples, granules were separated from the gelatin shell and kept in a thin and transparent glass tube. The glass tube was positioned in front of the laser source (three times each), and the Raman spectral data were recorded and compared with those of the other samples of the same brand or the authentic samples of the same brand. The concordance rate (match score) was calculated from the Pearson’s correlation coefficient between the test sample spectrum and the reference sample spectrum on a common interpolated wavenumber scale using the NuSpec Pro software (SciAps Inc., Laramie, WY, USA), and the Raman spectral data were input into the Unscrambler (CAMO Software, Oslo, Norway) for principal component analysis (PCA). The PCA model was constructed for three sets of samples, wherein each set represents five points, averaged from the 45 spectral data obtained from the different regions of each sample. Spectral data of the reference samples were used for the PCA model as the calibration set. The optimal number of principal components was determined from the internal cross-validation where authentic samples were treated as the reference set^47^. Spectral pre-processing involved the application of Savitzky-Golay smoothing and differentiation filter (second-degree polynomial and first derivative) to remove noise and baseline signals. We then performed unit-area normalization by applying Standard Normal Variate to the smoothed and differentiated signals^48–50^.

### Price

The prices of samples were recorded in local currency (BDT) and converted from local currency to US Dollar (USD), based on the exchange rate given by the money exchange office in Dhaka on April 09, 2018 (US$1 = Tk 83.5). Prices for the different strengths of medicine were calculated individually for each of the samples and expressed as the median unit price of an individual medicine; the observed individual prices were compared to an international reference price as recommended by the WHO/HAI manual^51^. The Median Price Ratio (MPR) was calculated by dividing the median unit price of an individual medicine by the median supplier prices from the Management Sciences for Health (MSH) 2015^52^.

### Statistical analyses

Statistical analyzes was performed using IBM SPSS Statistics for Windows, version 25 (IBM Corp., Armonk, NY, USA). The Fisher exact test was performed online (Extended—http://aoki2.si.gunma-u.ac.jp/exact/fisher/getpar.html). A significance probability of 1% was used for these analyses. Confidence intervals were calculated using descriptive statistical analysis. The criterion of significance was taken as p < 0.05. Means, standard deviations, and coefficient of variation (CV%) were calculated using Microsoft Excel 2016. Graphs for figures were generated using GraphPad Prism (version 9.0, GraphPad Software Inc., San Diego, CA).

## Results

### Sample description

A total of 189 samples were collected in this study. Samples were purchased by selecting at least one sample of either ESM, CFIX, or CVA-AMPC from the 210 outlets visited. From the list, 28 (13.3%) outlets had to be excluded as the shops were either non-existing or found closed at the time of sampling. Additionally, two ESM samples, two CFIX samples, and three CVA-AMPC samples were provided by the manufacturers upon request. All the collected samples were found to be domestically manufactured; 100 samples (54.9%) were collected from Dhaka North City Corporation area and the rest (45.1%) were collected from Dhaka South City Corporation. Samples from retail shops accounted for 78.6% of the total samples collected, whereas 20.9% samples were collected from wholesale shops selling retail medicine. Only one CFIX sample was collected from a shop inside a hospital facility. No provider asked to see a medical prescription from the buyers during the purchase of samples. Further details of the collected samples are summarized in Table 1.

**Table 1:** Outline of the samples collected and analyzed from Dhaka City Corporation, Bangladesh in 2018.

Medicine	Number of manufacturers (n)	Strength	Dosage form (n)	Number of samples	
				n	%
Esomeprazole (ESM)	22	20 mg	Enteric-coated tablet	16	8.5
			Enteric-coated capsule	48	25.4
Cefixime (CFIX)	18	200 mg	Capsule	60	31.8
		400 mg	Capsule	1	0.5
		200 mg	Tablet	1	0.5
Amoxicillin-Clavulanic Acid (CVA-AMPC)	5	625 mg (500 mg AMPC and 125 mg CVA)	Film-coated tablet	63	33.3
Total				189	100.0

### Results of observation

#### Shop observation

Samples were collected only from shops listed by the DGDA (no samples were collected from illegal shops). During sample collection, details regarding the storage conditions of the medicines were recorded. Among the visited shops, only 13 (7.2%) shops were found to be equipped with air-conditioning, although four of them were not functional; the remaining shops (92.8%) did not have air-conditioning. However, there was no significant temperature difference (p = 0.117) between shops with air-conditioning (30.1 ± 1.5 °C) and without air-conditioning (31.7 ± 2.5 °C). Although a significant humidity (relative humidity-RH) difference was observed (p < 0.05) between shops with air-conditioning (52.2% RH ± 8.7) and without air conditioning (59.6% RH ± 7.9).

#### Sample observation

Samples were checked for 46 points according to the FIP Tool for Visual Inspection of Medicines^38^. Overall, packaging analysis of the samples was satisfactory. One ESM sample had two different batch numbered strips from the same secondary packaging box (D-106). Two samples (A-102, B-213) did not have an insert inside the box, one of which (B-213) was confirmed to be falsified after chemical analysis. One CVA-AMPC sample (C-308) was found with a spelling error in the insert regarding the strength of the product (Supplemental Fig. S2). Although CVA-AMPC is usually film coated, no film coating was observed on the tablets from this sample when opened during analysis. The tablet surfaces were also dirty with speckles (Supplemental Fig. S2). The sample was also suspected to be falsified after chemical analysis.

### Authenticity, legitimacy investigation, and registration verification

The names and addresses of manufacturers and wholesalers were identified through the product packages and manufacturer websites. Questionnaires were sent to the respective manufacturers of ESM, CFIX, and CVA-AMPC by e-mail in July 2018, and 5 manufacturers out of 33 gave their response by 04 September 2018. Forty samples (21.2%) out of 189 were confirmed as genuine products by four manufacturers, and no falsified samples were reported (Table 2). Most manufacturers have a contact e-mail address, but in many cases, we did not obtain a response, even after sending a reminder mail to non-responders. The results of our authenticity investigation are summarized in Table 2.

**Table 2:** Authenticity, legitimacy investigation, and registration verification of the samples and manufacturers.

Authenticity investigation by the manufacturers				
Generic	Authentic, n (%)	Not authentic, n (%)	Unknown, n (%)	Total, n
ESM*	14 (21.9%)	0 (0.0%)	50 (78.1%)	64
CFIX**	11 (17.7%)	0 (0.0%)	51 (82.3%)	62
CVA-AMPC***	15 (23.8%)	0 (0.0%)	48 (76.2%)	63
Total, n (%)	40 (21.2%)	0 (0.0%)	149 (82.8%)	189 (100.0%)

Legitimacy verification by DGDA				
Generic	Legitimate	Illegitimate	Unknown	Total, n
ESM^§^	20 (91.0%)	1 (4.5%)	1 (4.5%)	22
CFIX^¶^	17 (89.5%)	2 (10.5%)	0 (0.0%)	19
CVA-AMPC	5 (100.0%)	0 (0.0%)	0 (0.0%)	5
Total, n (%)^†^	42 (91.3%)	3 (6.5%)	1 (2.2%)	46 (100.0%)

Registration verification by DGDA				
Generic	Registered	Unregistered	Unknown	Total, n
ESM^a^	61 (96.8)	2 (3.2%)	0 (0.0%)	63
CFIX	60 (96.8)	2 (3.2%)	0 (0.0%)	62
CVA-AMPC	63 (100.0)	0 (0.0%)	0 (0.0%)	63
Total, n (%)	184 (97.9%)	4 (2.1%)	0 (0.0%)	188 (100.0%)

*Two esomeprazole samples were provided by the manufacturers upon request; **Two CFIX samples were provided by the manufacturers upon request; ***Three amoxicillin-clavulanic acid samples were provided by the manufacturers upon request; ^§^One manufacturer of esomeprazole did not have manufacturing or marketing authorization to produce and sell esomeprazole capsule; ^¶^One manufacturer was not listed as a registered manufacturer of CFIX and manufacturing license was cancelled for another manufacturer by DGDA but their samples were circulating in the market and one of their sample was identified as falsified; ^†^Combining all samples total number of manufacturers were n = 33; a One esmeprazole sample was provided by AstraZeneca which was used a standard esomeprazole sample.

Although questionnaires were sent to the DGDA by e-mail in July 2018, we initially received no response. According to our first stage of registration verification, fifty-one out of 62 ESM samples (85%) were verified for registration from the DGDA drug product registration list in their website. Eight (13.3%) samples could not be verified, as the registration numbers in the package were different to the DGDA registration number. While one sample (B-114) was registered for tablet dosage form, we collected capsules from this manufacturer with the same registration number. In case of CFIX samples, n = 47 samples (78%) could not be verified due to differences in the registration number on the package and in the DGDA list. Moreover, one manufacturer was not listed as a registered manufacturer of CFIX raising legitimacy concerns. For CVA-AMPC, 52 out of 60 samples (86.7%) from three manufacturers had different registration numbers from those in the DGDA list and therefore could not be verified initially. At the beginning of 2020, DGDA crosschecked the registration status of the collected samples and confirmed that some newly changed registration numbers had not been updated on their site. However, two manufacturers were identified who were not licensed to produce CFIX. Another manufacturer was only licensed to produce a tablet dosage form of CFIX, although a capsule dosage form from this manufacturer was available on the market. The results of our registration verification of the samples and manufacturers are summarized in Table 2.

### Results of chemical analysis

Among all the tested samples, 18 samples (0.095; 95% CI 5.3–13.7) were found to be non-compliant after the final stage of at least one of the pharmacopoeial tests. Among the non-compliant samples, two samples were identified as falsified medicine (CFIX, 1; CVA-AMPC, 1). Raman spectroscopy and HPLC analysis of the falsified samples confirmed the absence of any APIs. The remaining 171 samples (90.5; 95% CI 86.3–94.7) were compliant with all pharmacopoeial tests according to the declared pharmacopoeia, i.e., USP or BP. The results of our chemical analyses are summarized in Table 3.

**Table 3:** Results of chemical analyses of all samples.

Generic	Test	No. of samples, n	Tested, n	Compliant, n (%)	Non-compliant, n (%)
ESM^†^	Potency	64	64	64 (100.0%)	0 (0.0%)
	Content uniformity	64	64	57 (100.0%)	0 (0.0%)
	Dissolution	64	64	Acid stage: 64 (100.0%); Buffer stage: 57 (89.1%)	Acid stage: 0 (0.0%); Buffer stage: 7 (10.9%)
	All test	64	64	57 (89.1%)	7 (10.9%)
CFIX^‡^	Potency	62	62	57 (91.1%	5 (8.1%)
	Content Uniformity	62	62	56 (90.3%)	6 (9.3%)
	Dissolution	62	62	59 (95.2%)	3 (4.8%)
	All test	62	62	55 (88.7%)	7 (11.3%)
CVA-AMPC^§^	Potency	63	63	60 (95.2%)	3 (4.8%)
	Content Uniformity	63	63	60 (95.2%)	3 (4.8%)
	Dissolution	63	63	61 (96.8%)	2 (3.2%)
	All test	63	63	59 (93.7%)	4 (6.3%)
All samples^¶^	Chemical analysis	189	189	171 (90.5%)	18 (9.5%)

^**†**^Three esomeprazole samples did not undergo all stages of pharmacopoeial analysis due to limited number of units**; **^‡^Two CFIX samples did not undergo all stages of pharmacopoeial analysis due to limited number of units**; **^§^Three CVA-AMPC samples did not undergo all stages of pharmacopoeial analysis due to limited number of units**; **^¶^Samples that did not go all stages of pharmacopoeial test were mostly authentic samples provided by the manufacturers upon request.

**Figure 1:**
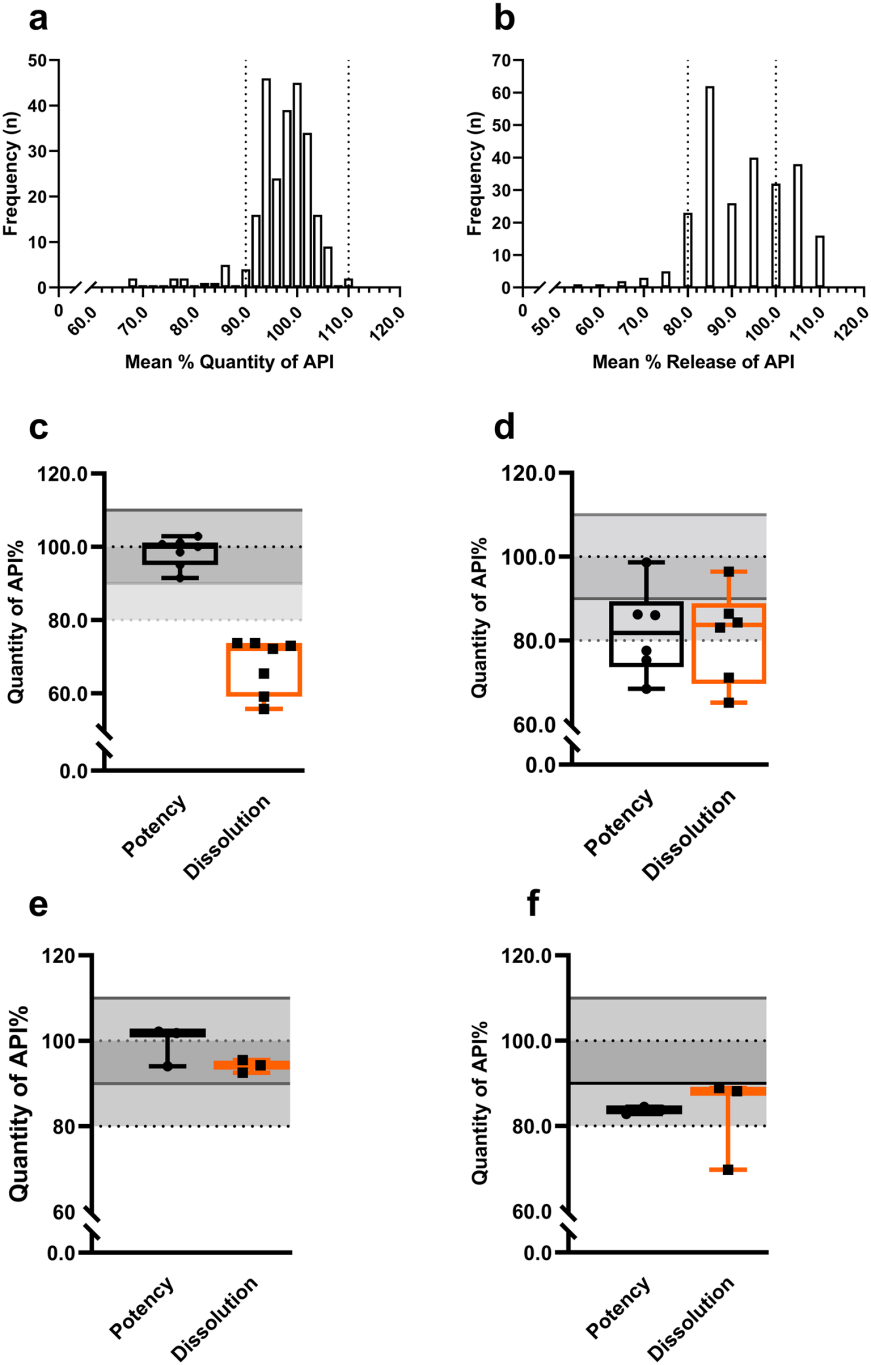
Frequency distribution of the mean quantity of samples in the potency and dissolution tests along with the potency versus dissolution rate of non-compliant samples. (**a**) Frequency distribution of the mean percent quantity of API for all samples. Dotted line represents the 90.0 to 110.0% cut-off for tolerance limit for ESM & CFIX, and 90–120% cut-off for tolerance limit for CVA-AMPC; (**b**) Frequency distribution of mean percent release of API for all samples in the dissolution medium. Dotted line represents the Q value cut-off for mean percent dissolution rate of ESM (75.0%), CFIX (75.0%), AMPC (85.0%), and CVA (80.0%); Figure (**c**–**f**) mean quantity versus mean dissolution rate found in non-compliant samples of ESM, CFIX, AMPC, and CVA-AMPC, respectively. The plot represents the minimum to maximum values of the samples, and individual samples in the potency test are indicated with a round maker and individual samples in the dissolution test with a square marker. The shaded area in the plot with a solid border line represents the cut-off for their acceptance limit in the potency test, and the shaded area in the plot with a dashed border line represents the cut-off for their Q value in the dissolution test (as explained above).

The average quantity of all samples compliant in the potency test was 100.1% ± 4.7 (95% CI 99.4–106.0) with a minimum mean of 90.1% API and a maximum mean of 111.8% API. The frequency distribution chart of all samples in the potency test is shown in Fig. 1a. In contrast, the average quantity of all the non-compliant samples in the potency test was 78.8% ± 7.1 (95% CI 73.7–83.8). Among the non-compliant samples, we identified samples with a minimum mean potency of 68.5% and a maximum mean potency of 86.2%.

High inter-unit variability was observed for nine (4. 8; 95% CI 1.7–7.8) of the total collected samples. Notably, among the non-compliant samples in the content uniformity test, none were from the ESM or amoxicillin samples.

In the dissolution test, the average percent release of API in the dissolution medium within the specified time was 93.3% with ± 9.5 (95% CI 92.1–94.5) for all the samples meeting pharmacopoeia acceptance criteria. The lowest amount of API released in the medium after the pharmacopoeia specified time was 71.2% and the maximum was 112.5%. The frequency distribution chart of all samples in the dissolution test is shown in Fig. 1b. Conversely, the mean percent release of API in the samples failing to meet the pharmacopoeia specified criteria in the dissolution test was 70.3% ± 10.0 (95% CI 63.6–76.9). The mean percent release in those samples was observed to be 55.7%, with a maximum of 93.4%. Surprisingly, all of the ESM tablets and granules kept their integrity during the acid stage of the dissolution test. Nevertheless, the dissolution rates of some samples were low in the buffer stage of the dissolution test, releasing less ESM than the pharmacopoeia specified range (Fig. 1c). Among the non-compliant samples, one ESM sample (B-103) demonstrated extreme failure, as two units showed as low as 1.3% release of API in the medium in the second stage of the dissolution test. Mean Quantity of the API of non-compliant samples versus their mean dissolution rate in the dissolution test are presented in Fig. 1c–f. As demonstrated in Fig. 1e and f, the potency and dissolution rate of AMPC in the CVA-AMPC samples were within the compliance range. Nevertheless, they were non-compliant due to the lower content of CVA in the co-amoxiclav samples. In spite of having lower potency than the declared amount, a comparatively higher dissolution rate was observed for CVA (Fig. 1f).

### Raman scattering analysis coupled with match score and PCA

**Figure 2:**
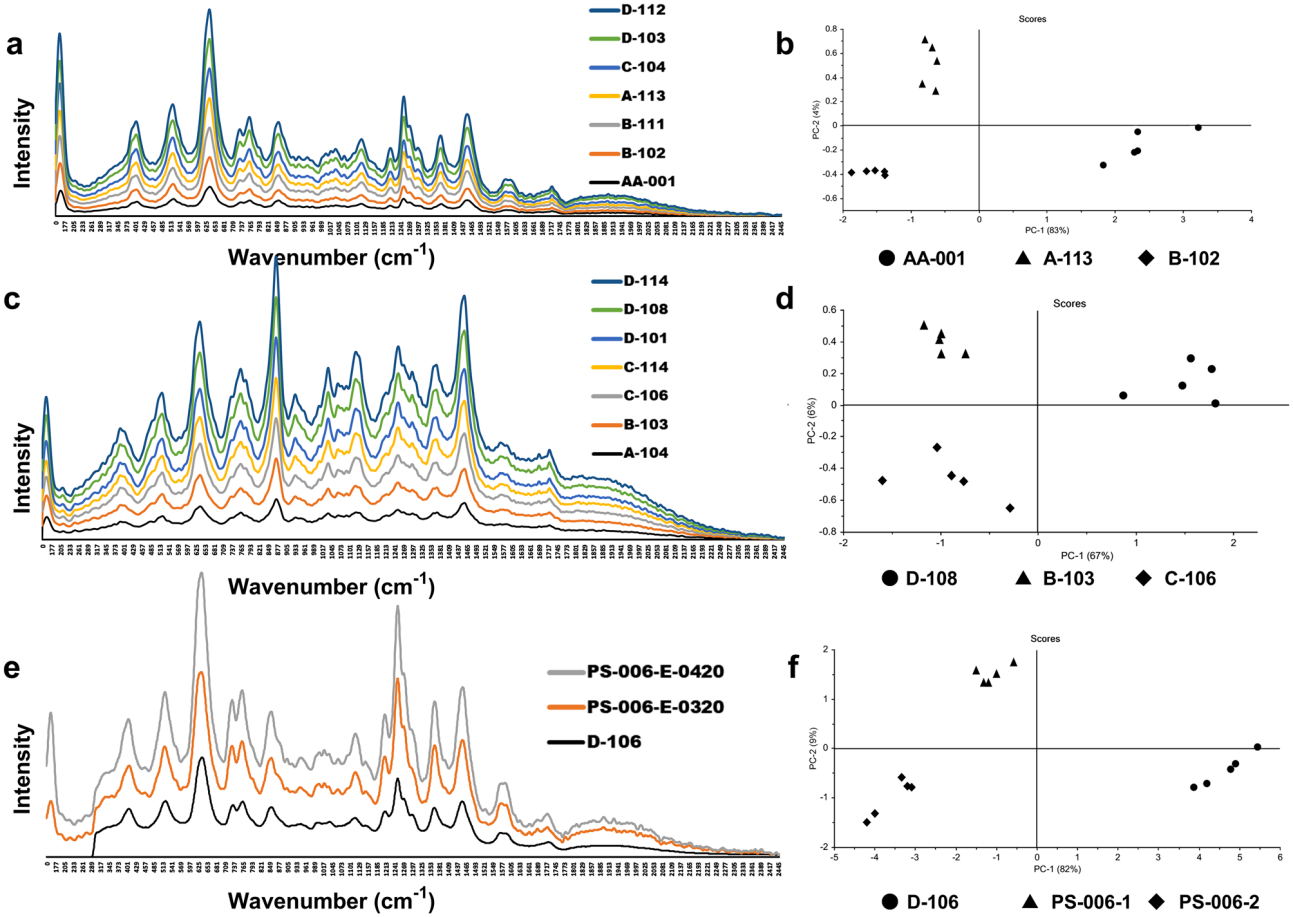
Raman spectra and principal component analysis (PCA) score plot of the compliant and non-compliant ESM samples in dissolution tests from the same manufacturers. Spectra were rescaled for clarity and overlaid without changing the plane or baseline shifting. Raman spectra were processed with smoothing and normalizing for the PCA score plot. (**a**) Raman spectra obtained from compliant (AA-001, A-113, D-103, and D-112) and non-compliant (B-102, B-111, and C-104) samples of manufacturer ‘X’. Differences were observed between the samples in both HPLC and the Raman spectra, despite the sample being from the same manufacturer. Match scores between sample AA-001 (authentic sample provided by the manufacturer) and A-113, AA-001, and B-102, and A-113 and B-102 were 97%, 95%, and 96% respectively; (**b**) PCA score plot of the representative samples AA-001, A-113, and B-102 in the PC1–PC2 plane. PC-1 accounts for 83% of the variance in the data, while PC-2 accounts for 4% explaining 87% of the total variance. According to the chosen separation distance (from the origin), it was possible to identify at least two clusters, one with samples showing poor dissolution rate in the buffer stage and another with samples releasing required amount of drug; (**c**) Spectra obtained from the compliant (A-103, A-104, C-114, D-101, and D-114) and non-compliant (C-106 and D-108) samples of manufacturer ‘Y’. Observed match scores between B-103 and C-106, B-103 and D-108, and D-108 and C-106 were 96%, 95%, and 93%, respectively; (**d**) PCA score plot of the representative samples B-103, C-106, and D-108 in the PC1–PC2 plane. PC-1 accounts for 67% of the variance, while PC-2 accounts for 6% thus explaining 73% of the total variance. Samples from manufacturer ‘Y’ projected in that PC space are distributed in two clusters with differences in the first and second PC directions; (**e**) Spectra obtained from the compliant (D-106) and non-compliant (PS-006) samples of manufacturer ‘Z’. As described in the text, sample PS-006 had two different types of strips with different batch numbers and expiry dates. Match scores between D-106 and PS-006-E-0320 (1), D-106 and PS-006-E-0420 (2), and PS-006-(1) and PS-006-(2) were 83%, 80%, and 86%, respectively; (**d**) The two groups of projected samples are separated mainly along the PC1 direction PC-1which accounts for 82% of the variance, while PC-2 accounts for 9% of the variance in the Raman spectra of those samples.

Raman spectroscopy was employed to explore the distinctive behavior between compliant and non-compliant samples, principally by match score and PCA. Raman spectra were analyzed after preprocessing and were subjected to PCA to investigate the similarity of chemical components between and/or among the samples. In most of the cases, non-compliant samples were compared against the authentic samples, if available. Otherwise, collected compliant samples from the same manufacturer were chosen for comparison. In the case of ESM samples, some manufacturers’ samples showed variances in chemical analysis, where some samples of the same brand were compliant while others were not. The results were further confirmed by Raman analyses and their PCA (Fig. 2). Compliant and non-compliant representative samples from those manufacturers showed a variation in the match score and a wide distribution on the score plot, suggesting significant differences among samples even though they were of the same generic brand. The correlation and variance among three different samples from the same manufacturer were analyzed using PCA on the basis of their compliance and non-compliance in the pharmacopeial analysis (Fig. 2a–d). Although a more number of representative samples could provide more precise evidence, a negative correlation was observed in PC1 between the compliant and non-compliant samples (Fig. 2b,d). In PC2, a positive correlation was observed between two non-compliant samples. As shown in Fig. 2e and f, another ESM sample (PS-006) showed variation in the strips and batch number in the same packaging, as discussed in the observational analysis results. Granules from both the strips showed a very poor dissolution rate (59.2% and 59.0%) in the dissolution medium. Notably, another ESM sample (D-106) manufactured by the same manufacturer was found compliant. To investigate whether the observed differences between the reference and test samples were not just because of the batch-to-batch variation, the dissolution profiles of the two reference samples (authentic samples obtained from the manufacturers) were analyzed together. The reference samples almost overlapped on the different PCs, indicating that the between-batch variation was negligible.

**Figure 3:**
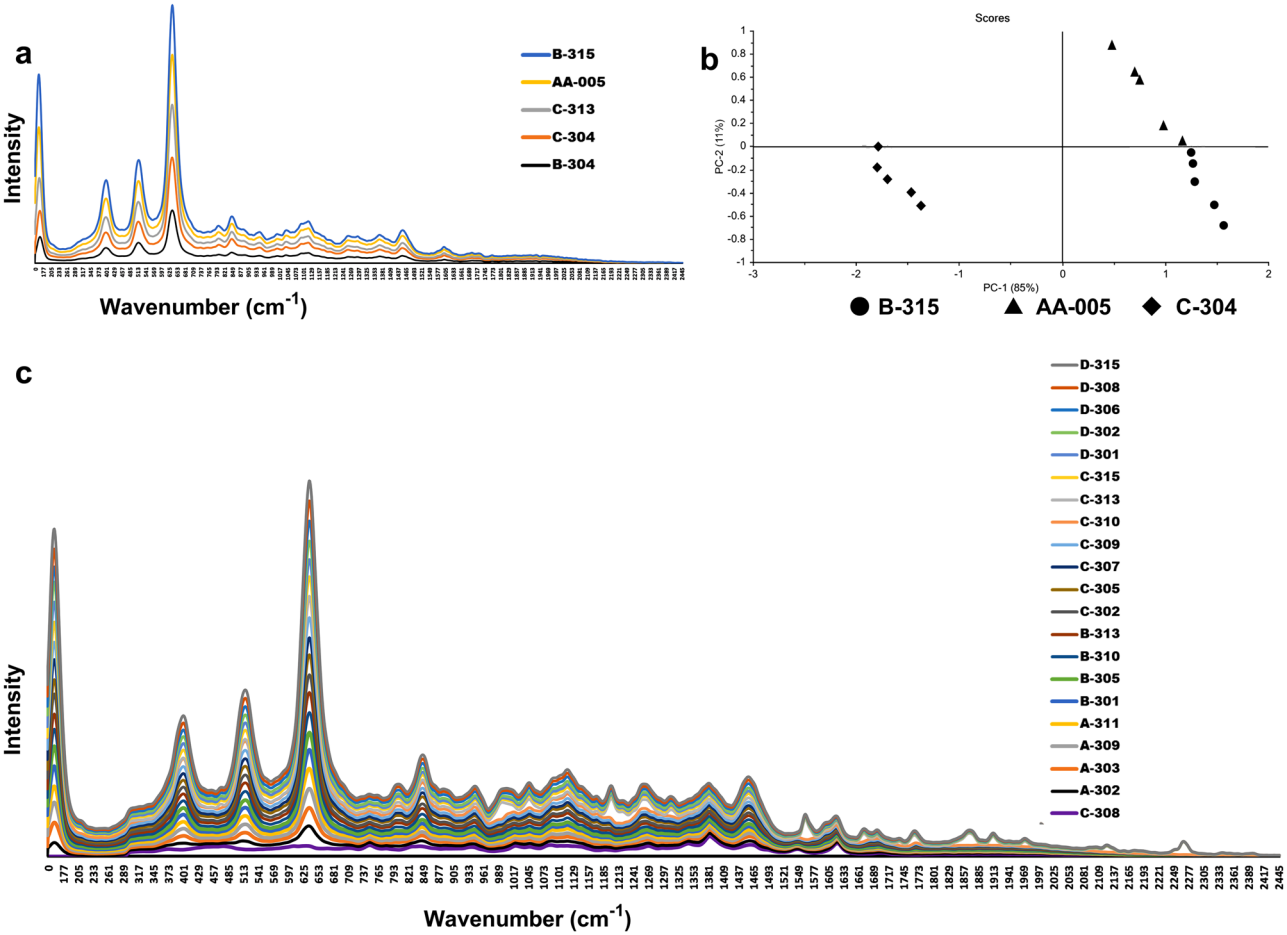
Raman spectra of CVA-AMPC samples from manufacturer A and B and PCA score plot derived from the Raman spectra. (**a**) Overlaid Raman spectra obtained from compliant (C-304 and C-313) and non-compliant (B-304, B-315, and AA-005) samples from manufacturer ‘A’. AA-005 was the authentic sample provided by the manufacturer, which was found non-compliant. Match scores between AA-005 and C-304, AA-005 and B-315, and between B-315 and C-304 were 89%, 99%, and 91%, respectively; (**b**) PCA score plot generated from the Raman spectra of representative samples AA-005, B-315, and C-304. Analysis of the samples by PCA, showed that the potency of the complaint samples differs from the other along PC1. The scores along PC1 correspond to their negative correlation coefficient with 85% variance, whereas non-compliant samples demonstrated a higher similarity rate among themselves with 11% variance in second PC plane; (**c**) Overlaid Raman spectra obtained from the samples of Manufacturer ‘B’. Among the n C-308 shared = 20 samples stated to be manufactured by them, all samples except a similar spectra.

Inconsistencies in the quality of the same generic brand samples from the same manufacturer were also observed in samples of CFIX and CVA-AMPC. In case of CVA-AMPC, though an authentic sample from a manufacturer failed to comply with the acceptance criteria, two samples out of four collected samples from this manufacturer were found to be compliant. Two authentic samples were separately provided by this manufacturer upon request. There is a possibility that any discrepancies in the physicochemical analyses between these samples may have occurred as a result of degradation of clavulanic acid resulting from the ineffective film coating (Sample B-315 in Fig. 3a and b; Fig. 3 in Supplemental File S2). Since the tablets in the samples from this manufacturer were packaged in strip, we assume that the chipping was caused by mechanical stress, and that it resulted in the permeation of moisture and consequent degradation of clavulanic acid^53–55^.

### Detection of falsified CFIX and CVA-AMPC

**Figure 4:**
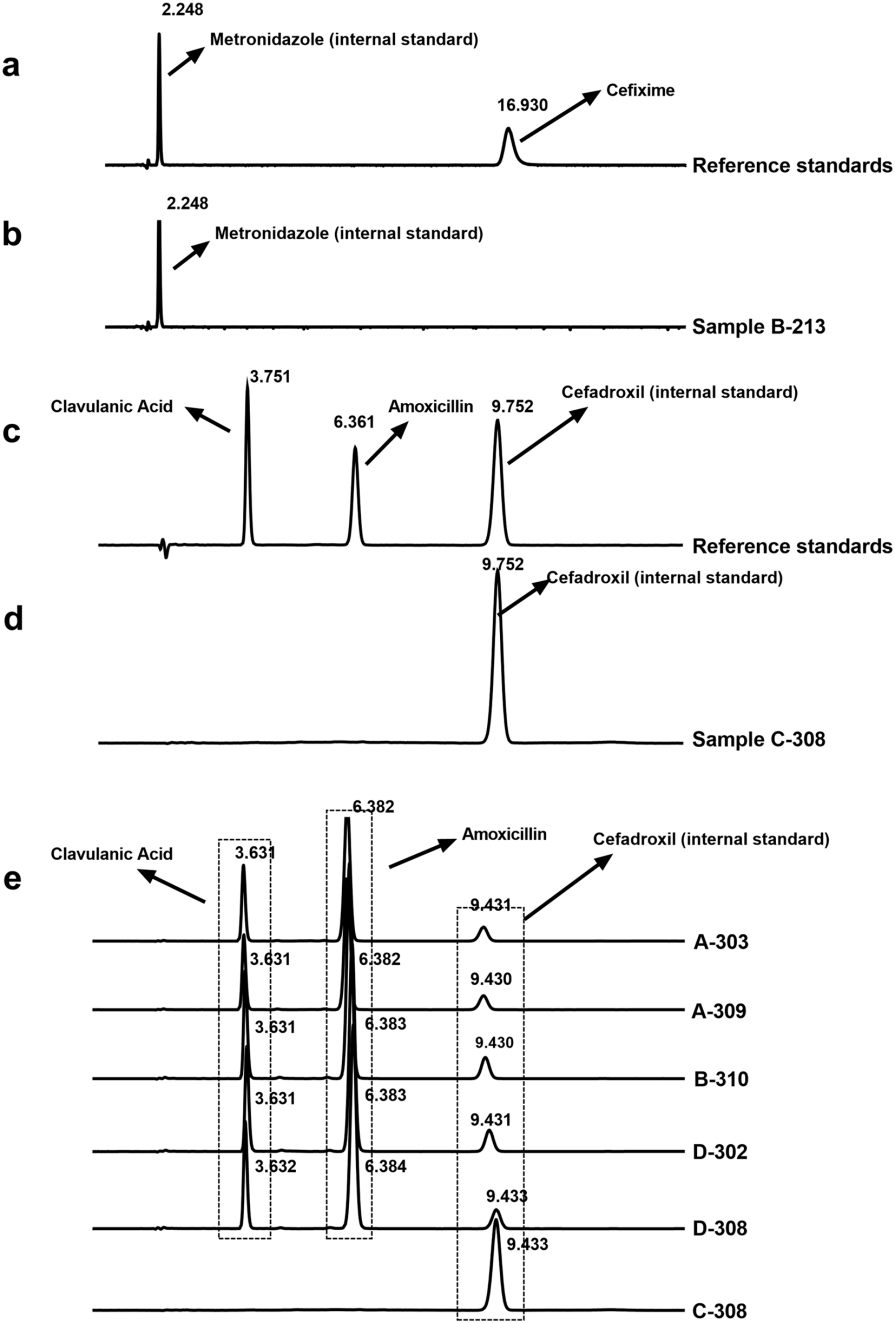
HPLC chromatogram demonstrating the photo-diode array detection of active ingredient in the standard and sample solutions. (**a**) Panel showing the chromatogram of the reference standard solution for the calibration curve, with metronidazole (the internal standard) at 2.248 min retention time (RT) and the reference standard at 16.930 min retention time; (**b**) Representative chromatogram of falsified CFIX sample B-213, showing only one peak at 2.248 min representing the internal standard (metronidazole added to the sample solution during analysis). No peak for CFIX was detected in this sample at 16.93 RT; (**c**) Representative chromatogram of the standard solution containing reference standard potassium clavulanate, reference standard amoxicillin trihydrate, and internal standard cefadroxil, with peaks at 3.751RT, 6.361RT, and 9.752RT, respectively; (**d**) Chromatogram of falsified CVA-AMPC sample C-308, showing only one peak representing the internal standard at 9.752 RT (cefadroxil added to the sample solution during analysis); (**e**) Overlaid chromatogram of several representative CVA-AMPC samples from the same manufacturer, where all the representative peaks appeared at same RT with reference standard solution.

**Figure 5:**
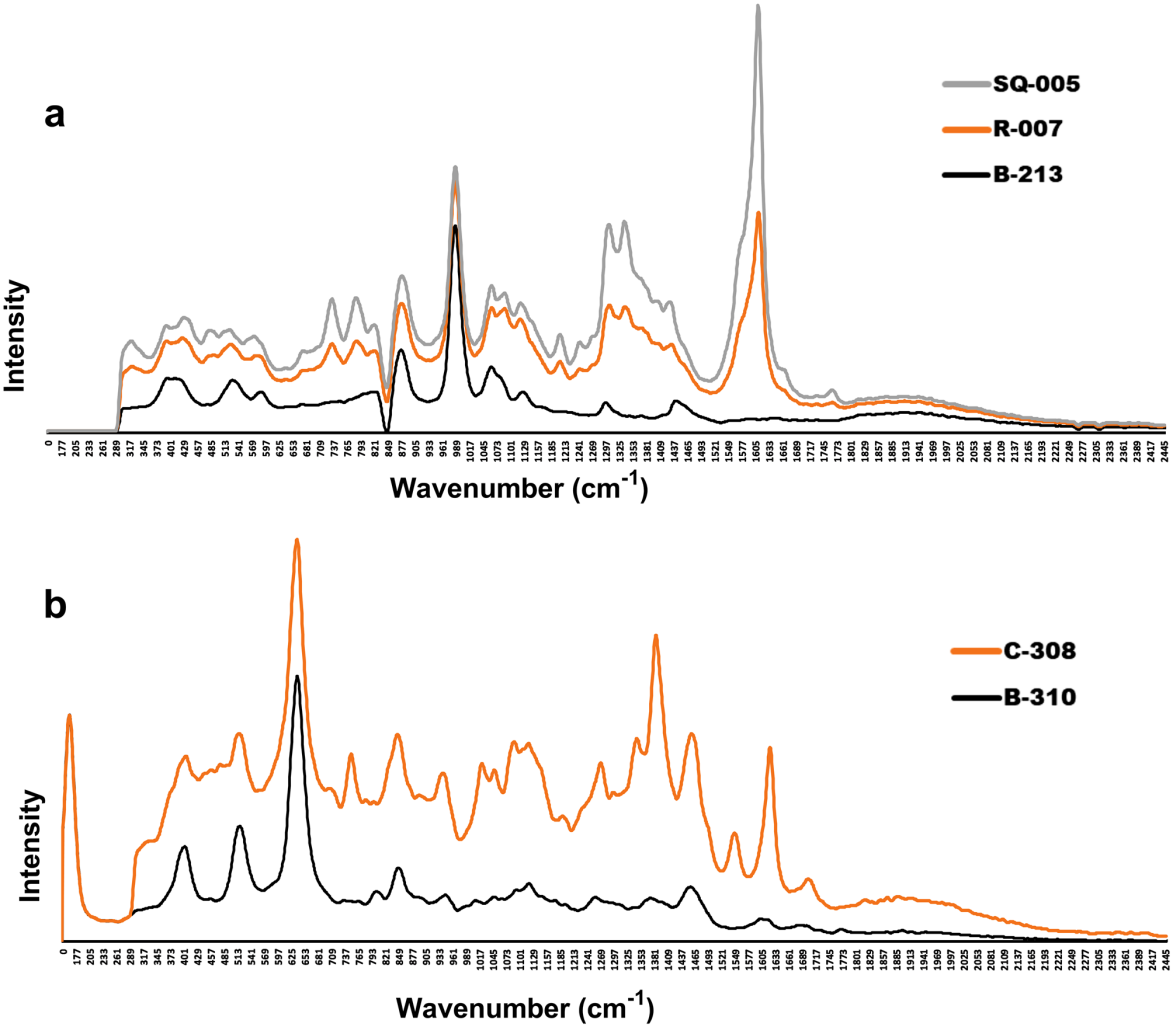
Raman spectra obtained from the falsified CFIX (B-213) and CVA-AMPC samples. Each spectrum represents the average of a set of 45 spectra. Five spectra were acquired from the different regions of the sample in solid form, and these were averaged. (**a**) Raman spectrum of falsified CFIX sample and two other reference samples obtained from two different manufacturers. Inset spectrum was acquired from the reference standard CFIX; (**b**) Raman spectra of falsified and reference CVA-AMPC sample obtained from the same manufacturer.

There was a reasonable level of agreement between the HPLC analyses and Raman spectroscopic analyses. In both the chromatographic and spectroscopic analyses, one CFIX and one CVA-AMPC sample appeared to be falsified (Figs. 4 and 5). The packaging labels of these samples stated that one contained CFIX and one contained CVA-AMPC, although no API was detected in either the assay or the spectroscopic analysis of these samples (see Sample B-213 in Figs. 4b and 5a; and Sample C-308 in Figs. 4d and 5b; Fig. 2 in Supplemental Fig. S2).

In case of the CVA-AMPC sample, the packaging was almost identical to the compliant samples from the same manufacturer. However, close observation of the packaging and insert showed a mistake in the insert (Fig. 2 in Supplemental File S2). For the CFIX sample, no comparator was available. To quantify the API, ten units of suspected CFIX sample and five units of suspected CVA-AMPC sample were assayed by HPLC, respectively. No chromatogram peak was observed with either of the samples (as described in Figs. 4 and 5). Additionally, 6 units of CVA-AMPC were used for the dissolution test that showed no disintegration of the drug in the medium (remained intact). Out of 20 collected CVA-AMPC samples that were manufactured by this manufacturer, one sample was found to be falsified. Presumably, the falsified sample had successfully mimicked the original. Further analysis of these samples was performed by Raman Spectroscopy, and the resulting spectra were markedly different from those of the reference standards and the other compliant samples (Figs. 3c and 5).

### Prices of medicines

**Figure 6:**
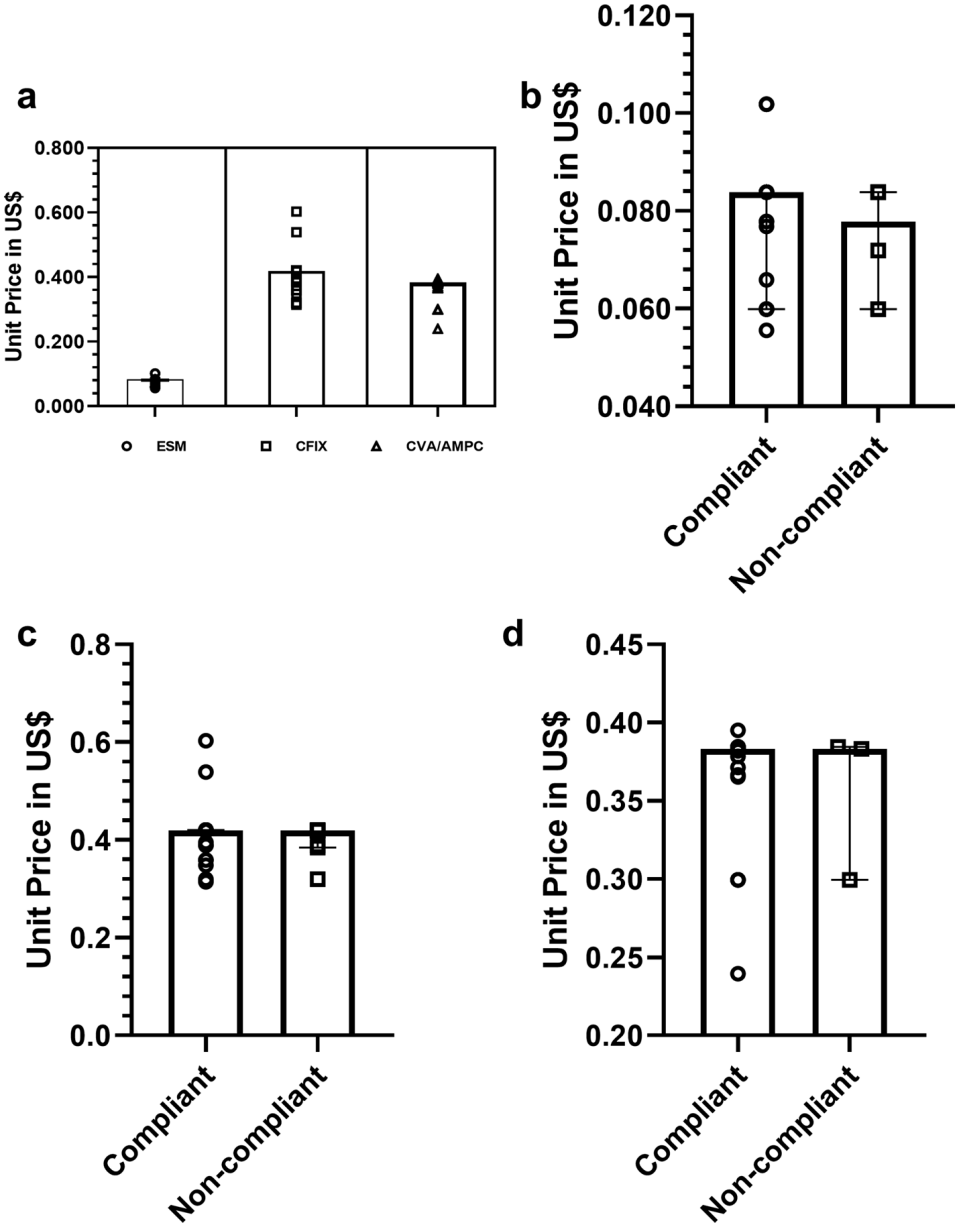
Unit price of medicines paid during sample collection. Price calculation did not include the price for n = 6 medicines as they were provided by the manufacturers. The plot represents all the values from lowest to highest paid price/unit expressed in USD along with the median price/unit. MPR relative to the IRP has been discussed in the text. (**a**) Unit price of ESM (n = 61), CFIX (n = 60), and CVA-AMPC (n = 60); (**b**) Unit price of compliant and non-compliant samples of ESM; (**c**) Unit price of compliant and non-compliant samples of CFIX, and; (**d**) Unit price of compliant and non-compliant samples of CVA-AMPC.

The discrepancy in price for all medicines was summarized by the minimum, 25th percentile, median, and maximum price values relative to the International Reference Price (IRP)^47^. The median unit price of a 20 mg ESM capsule was 0.084 ± 0.01 USD (minimum, 0.06 USD/unit; maximum, 0.102 USD/unit). The tablet formulation of 20 mg ESM had a median price of 0.06 ± 0.01/unit (minimum, 0.056; maximum, 0.078/unit). The price of a 20 mg ESM capsule was slightly higher than that of the 20 mg tablet, but not significantly. The Management Sciences for Health (MSH) price comparator was not available for ESM, although it’s MPR was significantly higher (p < 0.05) than that of omeprazole. Compliant ESM samples had a slightly higher price than the non-compliant ESM samples, although the difference was not significant (Fig. 6a,b).

The median price of 200 mg CFIX was 0.419 ± 0.04 USD (minimum, 0.314 USD/unit; maximum, 0.603 USD/unit) (Fig. 6a,c). The CFIX price was significantly higher than the MSH median price (*t*-test, p < 0.001). However, the MPR for CFIX was found to be 2.54. An MPR of 1 or less is commonly interpreted as efficient procurement in the public sector, while an MPR below 3 is considered acceptable for the private sector^56^. All of our samples were procured from private retail shops (the authentic samples were not included in the price calculations). One sample of CFIX was 400 mg and the price was 0.60 USD. The minimum price paid for 625 mg CVA-AMPC was 0.24/unit (maximum, 0.395/unit; median 0.383/unit), which was significantly higher than the MSH price (*t*-test, p < 0.001). For 625 mg CVA-AMPC, the MPR was found to be 2.35. No significant differences were observed between compliant and non-compliant samples of either CFIX or CVA-AMPC (Fig. 6a,d).

## Discussion

**Figure 7:**
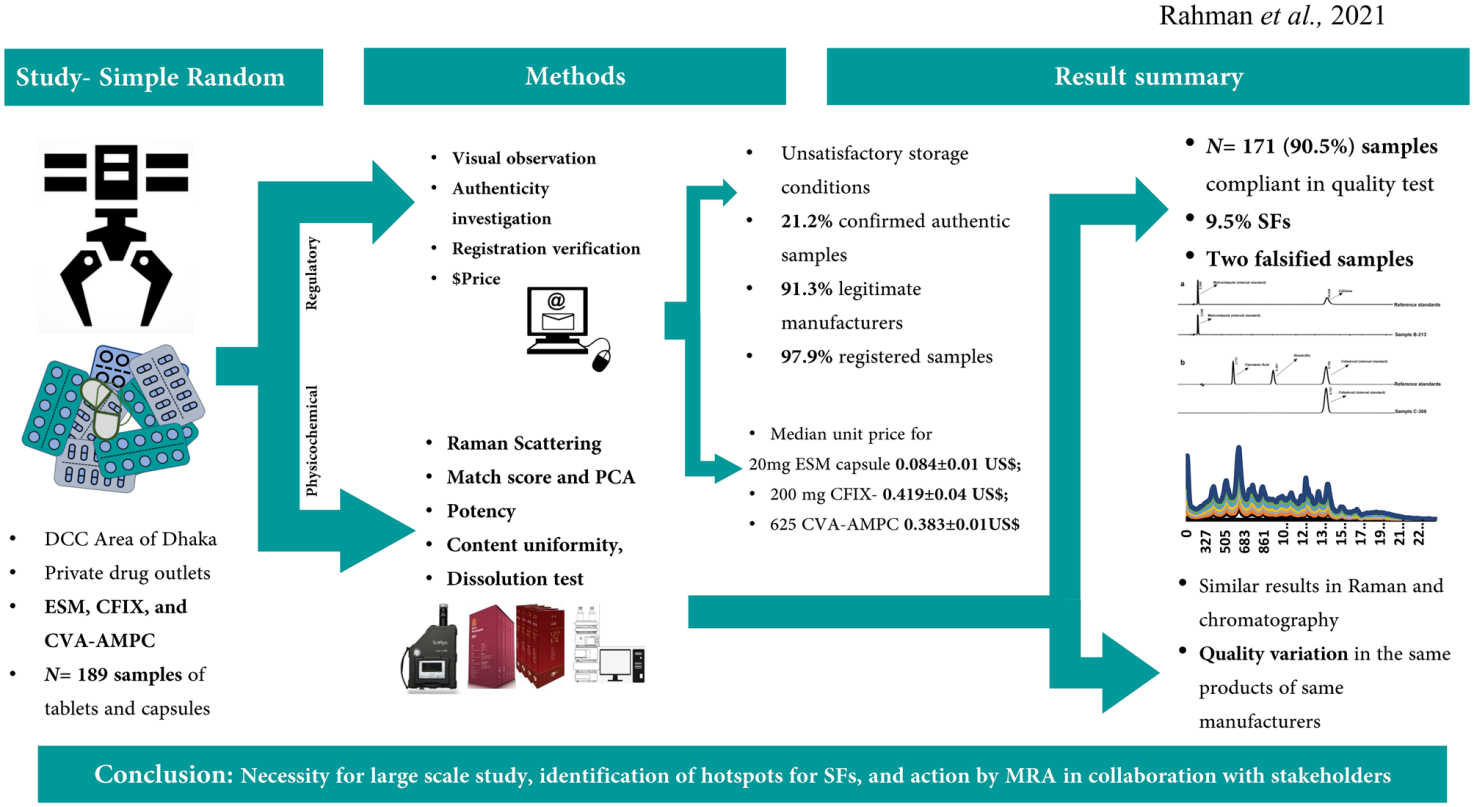
Study summary flow chart.

Recent research has revealed that an alarming percentage of analyzed medical products in LMICs were found to be unacceptable in terms of quality, with the additional concern that SF antibiotic could lead to drug resistance due under-dosing or no dosing at all^9,57,58^. It is important that tragic events like that reported in Bangladesh in 1995 are not repeated, especially in the Covid-19 pandemic era^21,59^. Despite achieving remarkable health and pharmaceutical improvements since gaining independence in 1971, adequate reporting of the quality of medicines using tested methodological practices has not been accomplished in Bangladesh^60^. This is the first comprehensive analytical study documenting the quality of selected medicines in private drug outlets in Bangladesh. A summary of the study has been presented in Fig. 7 as a flow chart.

The present study reports the analytical results from medicines for a non-communicable disease and two antibiotics, including one combination drug, as representative medicines. Overall, the quality of the majority of the samples analyzed was revealed to be good; more than 90% of the analyzed samples collected from the Dhaka City Corporation region complied with the pharmacopoeial reference ranges and were found to be of acceptable quality (Table 3). The results of this study are significant, when compared with the results of summarized studies conducted in other LMICs^9,56^. The WHO previously reported a failure of 19.5% (95% CI 18.8–20.3) of samples following random sampling, whereas Ozawa et al. reported that the prevalence of SF medicines ranged from 18.7% (95% CI 12.9–24.5) in Africa to 13.7% (95% CI 8.2–19.1) in Asia^9,57^. In contrast, the prevalence of substandard medicines in our study was found to be 8.5% (95% CI 4.5–12.4), and this was higher than the prevalence of falsified medicines 1.1% (95% CI − 0.4–2.5). In total, these results indicate a prevalence of 9.5% for SF medicines, lower than the assumed prevalence of 15% (p < 0.05; 95% CI 5.7–14.6).

While most of the samples were of good quality, 4.2% of samples (95% CI 1.4–7.1) contain less API than the stated amount in their packaging (Table 3). Another important observation was the uniformity of the content, with 4.9% of samples demonstrating high inter-sample variation; these were found to be non-compliant according to the ‘acceptance value (AV)’ set by the pharmacopoeia. The permissible range of AV values set by the pharmacopoeia was established to ensure that all individual units within a sample have a sufficient amount of the declared API. Indeed, it is essential that the patient receives a dosage close to that claimed in the label. However, in the cases reported above, it cannot be stated with certainty that every unit of the sample contained the exact same amount of API.

The drug dissolution test is a good predicator for the sample performance test, while allowing observation of physicochemical changes in tablets/granules in capsules^61–63^. The results presented in this study demonstrate that 5.3% samples (95% CI 2.1–8.6) were non-compliant (including all tested samples but excluding the falsified samples). The poor dissolution rates of the samples were a persistent problem, an observation which had previously been reported in our studies^60,61,64,65^. As an essential indicator of bioavailability and drug quality, the dissolution test has not been given as much attention as it deserves^62,66,67^. Despite the fact that all of the ESM samples contained the stated amount of API, a poor dissolution rate was observed in the buffer stage of the dissolution test in 10.9% of the ESM samples (Table 3; Figs. 1 and 4). HPLC analysis detected one inconsistent ESM sample, and this showed only a 1.3% dissolution rate of units in the second stage of the dissolution test. Unfortunately, a genuine comparator was not available for this sample to investigate further.

The reliability of the findings was further supported by the Raman spectroscopy results, which also confirmed the detection of two falsified medicines. The status of suspect samples identified during a visual observation using the FIP checklist was confirmed by the chemical analysis result (for example, sample D-106 appeared to be substandard and B-213 appeared to be falsified). However, packaging analysis alone could not detect additional falsified CVA/AMPA medicines (Figs. 4 and 5; Supplemental Fig. S2). Identical packaging from falsified samples could not be distinguished from that of the other samples stated to be manufactured by the same manufacturer, even after close observation of the packaging. In our study, handheld Raman spectroscopy was a practical and useful tool for the screening of suspicious samples, facilitating both identification of and characterization of suspicious samples (Figs. 2, 3, and 5). Samples showing different chromatographic analysis result but similar spectra could be differentiated using principal component analysis (PCA) of the Raman spectra (Figs. 2 and 3). One falsified sample of CFIX and one falsified sample of CVA-AMPC were initially identified using the handheld Raman spectroscopy device, demonstrating its usefulness for the detection of falsified samples. Hence, this comparatively low-cost device may prove a useful tool in field settings requiring quality analysis.

Despite the satisfactory results obtained in our chemical analyses, falsified medicines threaten the national supply and distribution chain (Figs. 3, 4, 5). No one test can serve as an ultimate tool for the detection of SF medicines, and the roles of MRAs and manufacturers remain in question, as the anticipated responses from the stakeholders regarding product authentication and legitimacy verification were below the expected level. In our authenticity investigation, a response was received from only five manufacturers, confirming the authenticity of 21.2% samples (Table 2). Better cooperation from the manufacturers in response to questionnaires is essential for any authenticity investigation. Furthermore, considering the export potential of pharmaceuticals, which is largely driven by domestic manufacturers, the governing authorities should focus on the active and effective regulatory surveillance of manufacturers.

Data from our study suggests that there is substantial scope for improving the storage situation of the distributed medicines, and for lowering the prices of the medicines in the private drug outlets. The observed sub-standard storage conditions for the medicines may be linked to degradation of the medicines, although no relationship could be established between storage and quality of medicines in our study^68^. As reported above, the prices for the collected medicines were slightly elevated relative to the international standards (Fig. 6), although the prices were not excessive. This suggests that the revised health policy intervention may be necessary to reduce the catastrophic out-of-pocket expenditure of patients, especially during long-term treatment.

The strength of this study is that it presents a comprehensive analysis of the collected samples including all stage of pharmacopoeial analysis for potency, a dissolution test using HPLC, and Raman spectroscopy combined with chemometrics. The sample size for this study was not large enough to allow prediction of the influencing factors and a comparative analysis. However, given the limited personnel resources, a larger sample size would limit the scope for such a detailed analysis. Indeed, longer processing times would make it difficult to complete the analysis before the expiry date of the collected samples. Finally, the study is not nationally representative and should be interpreted with caution, as samples were only collected from one urban area of the Capital City and illegal or unlicensed shops were excluded.

## Conclusion

The threat of SF medicines exists in Dhaka City Corporation, although the proportion of SFs was revealed to be relatively lower than the estimated proportion. In addition, differences in the quality of the same branded sample from the same manufacturer may put the patient at risk. Therefore, the national MRA, in an active collaboration with the manufacturers, should identify hotspots for these life-threatening poor-quality medicines, and take prompt and effective action. In our study, a consensus was observed between the portable Raman spectroscopy results and the observational and authenticity analysis results thus, implying that scope for these screening techniques should be considered in the field detection and evaluation of medicines. A full-scale analysis, including a dissolution test, is essential to accurately estimate the prevalence of substandard and falsified medicines.

## Supplementary Information

Supplementary Information.

Supplementary Figures.

## Data Availability

All relevant data available for publication are within the manuscript and the supporting information. Public sharing and distribution of raw data has been restricted by the Directorate General of Drug Administration (DGDA), Bangladesh.
