# Supplementary material for: A comprehensive analysis of selected medicines collected from private drug outlets of Dhaka city, Bangladesh in a simple random survey
Source: Sci Rep. 2022 Jan 7;12:234. doi: 10.1038/s41598-021-04309-1 (PMC8741935; doi:10.1038/s41598-021-04309-1)

A comprehensive analysis of selected medicines collected from private drug outlets of Dhaka city, Bangladesh, in a simple random survey

Mohammad Sofiqur Rahman*, Naoko Yoshida, Hirohito Tsuboi, James Regun Karmoker, Nadia Kabir, Simon Schaefermann, Yoshio Akimoto, Mohiuddin Ahmed Bhuiyan, Md. Selim Reza & Kazuko Kimura

Supplemental Figure S1: Location of sampling area. Location marker was formed from the GeoDASH (a geo-spatial data storing and sharing initiative by Bangladesh Government, https://geodash.gov.bd/maps/new) using the google base map

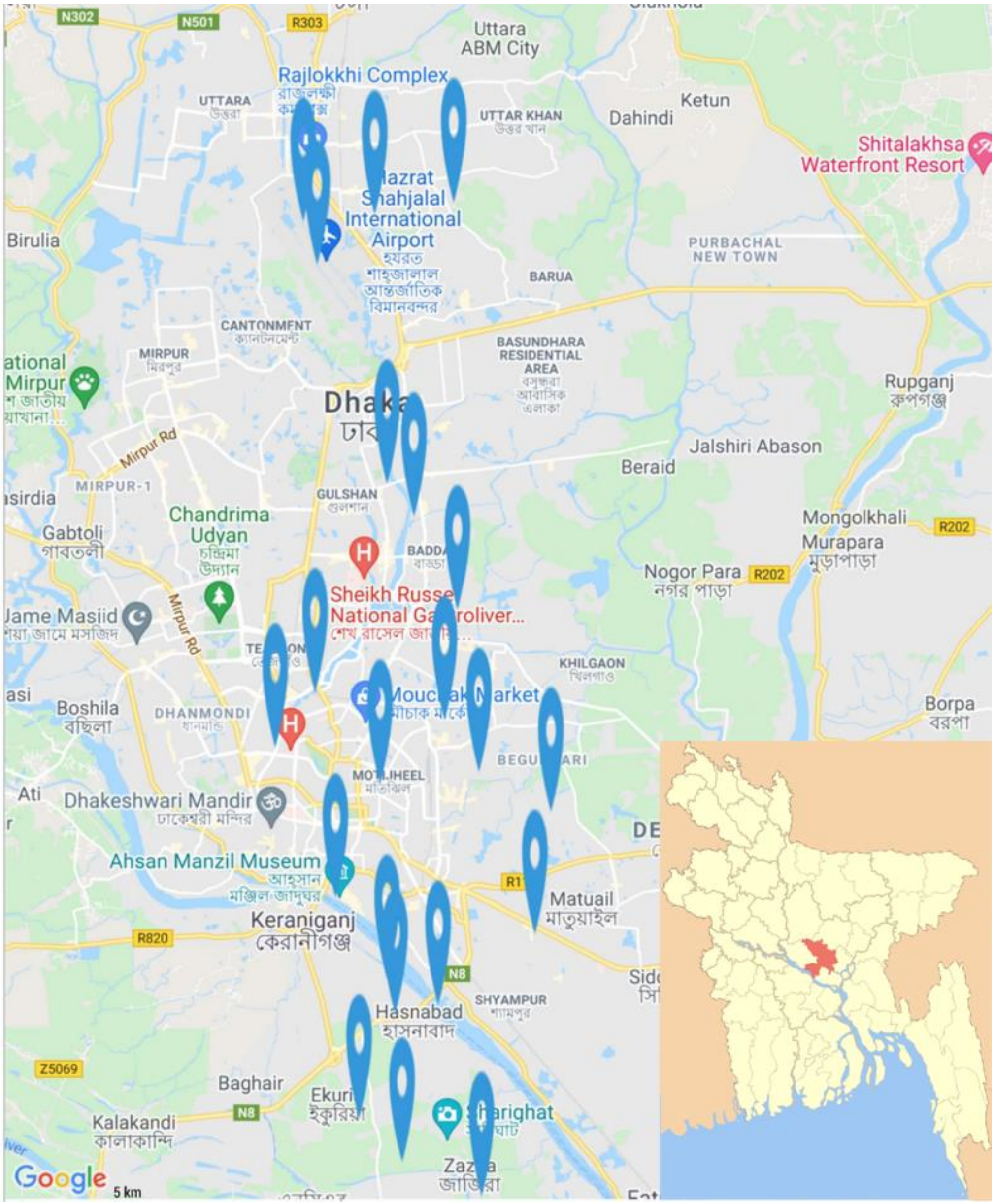

Supplemental Figure S2: Photograph of falsified samples and compliant sample. a) Falsified cefixime sample; b) Falsified amoxicillin-clavulanic acid sample; c) Spelling mistake in the insert; d) One representative compliant sample B-310 labelled as the same brand name and batch number, and e) Correct spelling in the insert of B-310

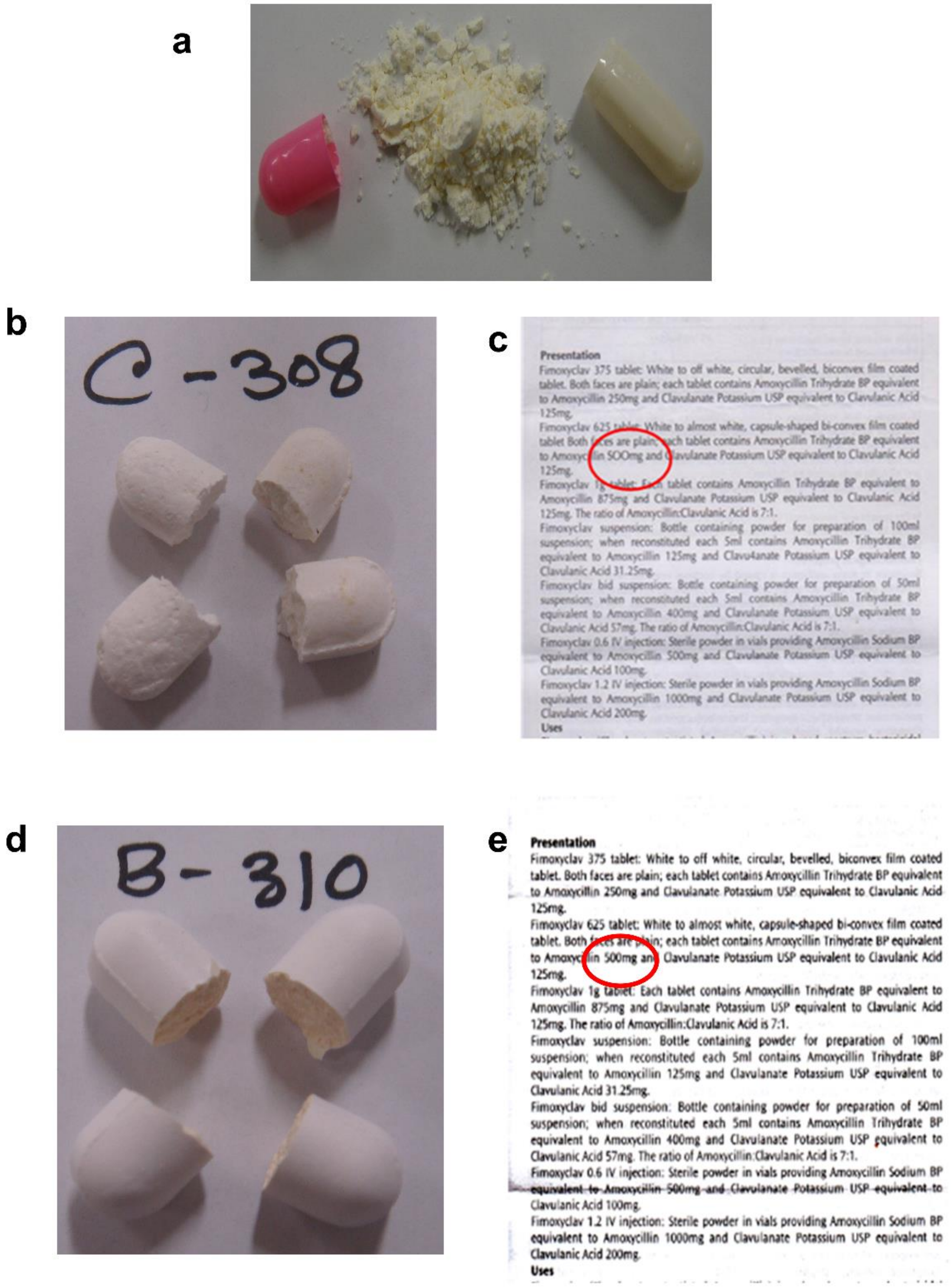

Supplemental Figure S3: Substandard CVA/AMPC sample (Sample B-315) with ineffective film coating.

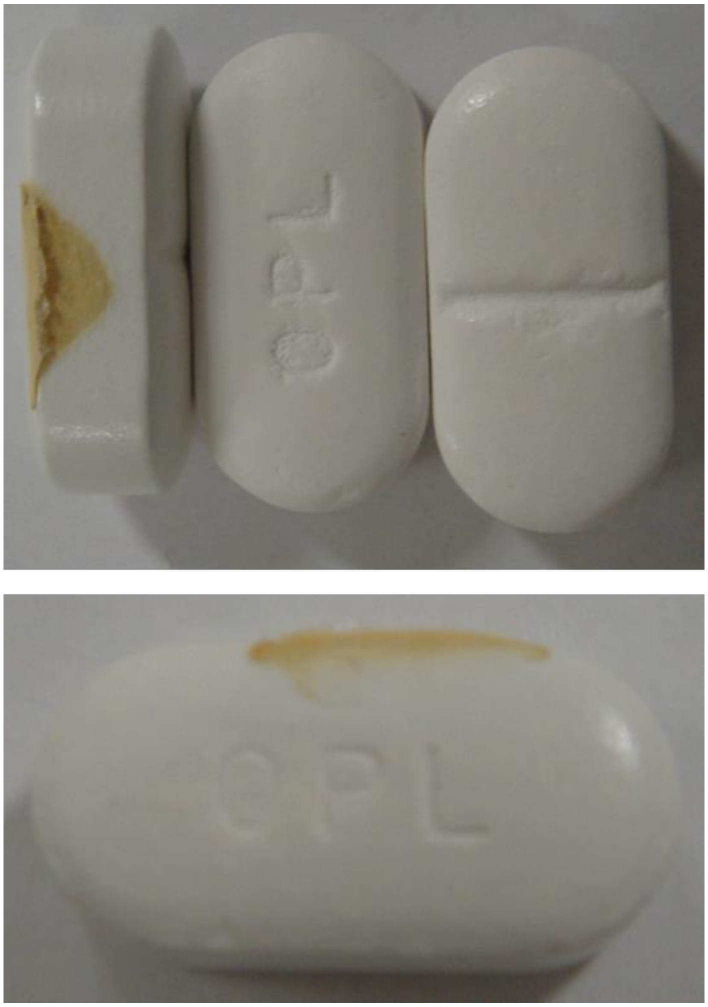

Supplement: Supplementary file 2 — Supplementary Figures. [file 41598_2021_4309_MOESM2_ESM.pdf]
